# Harnessing Youth Engagement at Faith Based Organizations to Combat Hypertension

**DOI:** 10.5888/pcd22.250115

**Published:** 2025-08-21

**Authors:** Rushabh H. Doshi, Pavan Khosla, Kanhai S. Amin

**Affiliations:** 1 Yale University School of Medicine, New Haven, Connecticut

Hypertension remains a significant public health challenge in the United States (1). According to the Centers for Disease Control and Prevention (CDC), nearly half of US adults (48.1%, 119.9 million) have hypertension or are taking antihypertensive medication (2). Despite its prevalence, hypertension is often asymptomatic, earning it the designation of a “silent killer.” As a result, nearly 16% of adults are unaware they have hypertension (3).

Despite this lack of symptoms, hypertension is easily and inexpensively detectable and is highly treatable; management significantly reduces risks of stroke, myocardial infarction, hypertensive retinopathy, nephropathy, and heart failure (4). Therefore, it is crucial that public health initiatives prioritize widespread screening to identify cases and connect individuals to appropriate care. Further, initiatives must simultaneously promote healthy behaviors among both hypertensive and disproportionately affected populations.

## Community Strategies to Combat Hypertension

Many community-based screening efforts against hypertension have emerged. These include health fairs at community centers, shopping malls, parks, and other locations to screen people and provide educational information (5). Mobile health units have also served as an avenue to bring screening services directly into underserved communities (6). However, in addition to substantial logistical and operational costs, these and other efforts are episodic — valuable for raising awareness but lacking repeat engagement. Other strategies such as barbershop programs, annual workplace screening programs, and pharmacy-based initiatives provide opportunities for multiple screenings but may also struggle to ensure effective behavior change and care coordination (7).

Alternatively, faith-based organizations, including churches, temples, and mosques, represent an underused yet promising avenue for hypertension screening and prevention, as noted by a recent systematic review (8). These religious institutions offer repeated interaction that not only reinforces health messaging but also fosters shared, locally informed strategies to connect members to primary care. For example, vulnerable communities may have shared learning on how to enroll in assistance programs or obtain care at local federally qualified health centers. However, we propose an augmentation to these typical faith-based initiatives by mobilizing youths, particularly adolescents, within these communities to lead the efforts. The purpose is multifold: cultivating leadership and healthy habits in youths while delivering screenings and health education in a way that resonates with members of the community and keeps them engaged. The innovation lies in the empowerment of youths as agents of change in their communities.

Youths in religious institutions can be trained to use automated blood pressure devices, which require minimal technical expertise. Unlike sporadic health fairs, youth-led programs can facilitate consistent screening opportunities after worship services, during community meals, or at congregation events. Regular engagement allows for multiple touchpoints, increasing the likelihood of identifying people with elevated blood pressure who might otherwise remain undiagnosed. Youths may also encourage people to reach out to primary care or facilitate the connection, lead brief educational presentations designed by expert dieticians, organize gardening events or walking clubs, or a plethora of other activities that can improve cardiovascular health.

This solution aligns with the core values of faith-based organizations, as many religious traditions emphasize stewardship of one’s body, care for community, and service to others. Youth leaders can contextualize health messaging within spiritual teachings, making prevention strategies more culturally resonant and effective. Additionally, pastoral support, such as health-focused sermons, can further reinforce health-promoting behaviors among congregants.

The approach addresses several limitations of traditional screening models. Unlike mobile health units, faith-based programs leverage existing infrastructure and community gatherings, reducing operational costs and logistical barriers. Second, it embeds follow-up mechanisms. Youths can serve as health ambassadors, personally reminding people with elevated blood pressure readings to seek medical care, adhere to prescribed treatments, and to have healthier lifestyles. Ultimately, the youths create accountability and actionability by following up with their community members.

Further, the intergenerational dynamics of this model creates unique benefits. Adults may demonstrate greater receptivity to health interventions led by youths in their communities. Parents, grandparents, and other community members hesitant to participate in screening at a health fair may engage more readily when invited by familiar youths within a trusted setting. These community members may also be more willing to listen to presentations on the effects of food and sodium on blood pressure that are led by youths from their communities at the end of a sermon. This may increase participation and engagement rates.

Additionally, exposure to health care may inspire career interests in medicine, nursing, or public health in the youths, helping to address diversity challenges in health care. Further, youth participants will gain health literacy, leadership abilities, and confidence in public speaking and interpersonal communication while learning how to lead healthier lives themselves.

## Enabling Success

Faith-based organizations hold deep-rooted trust within communities, particularly in areas where medical mistrust is prevalent (9). This trust can help dispel health misinformation, promote preventive screenings, and encourage healthy behaviors related to diet and exercise. To ensure program efficacy, congregations should identify health care professionals within their membership to serve as program champions and clinical advisors to the youths. These mentors can ensure use of proper screening techniques and proper interpretation of results, and they can develop protocols for managing elevated readings.

When congregations lack internal health care expertise, partnerships with nearby health care institutions can substitute to provide training and support. However, unlike initiatives led by members of the community, these outside groups would have to build trust with communities that have often been wronged by the health care establishment. For example, physicians have previously run screening fairs at churches in marginalized communities to find patients for lucrative but often not clinically warranted procedures (10).

To build trust in communities and run health initiatives, we believe that medical students and college students are particularly untapped resources. From our experience with health initiatives in New Haven, parents and religious communities welcome students into their organizations to serve as mentors to their youths. The purpose of these students would be to provide mentorship and volunteering rather than conducting a research study or predatory screenings. For youths in marginalized communities, this mentorship may be invaluable. We believe many college and graduate students would be interested in such a program. For example, college students already volunteer through the Hypertension Awareness and Prevention Program at Yale to screen people at soup kitchens for hypertension before facilitating their care at the Haven Free Clinic, a clinic run by medical students (11).

## Overcoming Barriers to Implementation

While faith-based, youth-driven hypertension screening programs hold promise, several challenges must be addressed. Congregations vary in size, resources, and youth engagement. Some may have well organized youth ministries that already run service projects while others may have limited organized youth activities. Further, some faith-based organizations may have less engagement from older youths who would be crucial in running more intricate parts of the initiative. Here, creating a governance structure that includes youths, health professionals, and faith leaders can distribute responsibilities, ensure dependability, and maintain momentum.

Another challenge is motivation and retention. The church leadership, youths, and parents of the youths must stay engaged throughout. Initially, developing educational materials for community members may be a barrier, but religious institutions can make use of existing information from the American Heart Association and the Centers for Disease Control and Prevention. Additionally, sustained long-term engagement can be ensured by celebrating people who successfully managed their hypertension.

Cultural sensitivity also plays a significant role. Religious congregations can be extremely diverse, and effective health-promotion messages must be mindful of cultural dietary preferences, language nuances, and moral or religious perspectives on health and body image. Collaborating closely with faith leaders in message development helps ensure health interventions align with religious teachings and traditions.

While these initiatives do not constitute formal medical care, safeguards must be in place to protect each person’s health information. Youths must be trained to respect privacy, and programs should establish clear protocols on data handling, including not recording or storing blood pressure readings without consent. While less ideal because it prevents tracking of follow-up and care coordination to local clinics, a program could also be implemented such that no data are logged and that participants are instead encouraged to use self-tracking sheets.

## Conclusion

Harnessing youth engagement within faith-based organizations presents a novel and sustainable approach to hypertension prevention and management. By integrating routine screenings into faith-based gatherings, empowering youths as health advocates, and leveraging the trust inherent in religious communities, this model has the potential to enhance early detection, foster intergenerational health awareness, and inspire future public health leaders. With strategic partnerships and culturally tailored messaging, this initiative could serve as a replicable framework for addressing other chronic disease challenges in community settings.
